# Impact of Soil Composition and Electrochemistry on Corrosion of Rock-cut Slope Nets along Railway Lines in China

**DOI:** 10.1038/srep14939

**Published:** 2015-10-09

**Authors:** Jiao Chen, Zhaoqiong Chen, Yingwei Ai, Jingyao Xiao, Dandan Pan, Wei Li, Zhiyu Huang, Yumei Wang

**Affiliations:** 1 Key Laboratory of Bio-resources and Eco-environment (Ministry of Education), College of Life Sciences, Sichuan University, Chengdu 610064, China; 2 Chengdu Medical College, Chengdu 610500, China; 3 Institute of Mountain Hazards and Environment, CAS, Chengdu 610041, China

## Abstract

Taking the slope of Suiyu Railway to study, the research separately studied soil resistivity, soil electrochemistry (corrosion potential, oxidization reduction potential, electric potential gradient and pH), soil anions (total soluble salt, Cl^−^, SO_4_^2−^ and 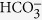), and soil nutrition (moisture content, organic matter, total nitrogen, alkali-hydrolysable nitrogen, available phosphorus, and available potassium) at different slope levels, and conducted corrosion grade evaluation on artificial soil according to its single index and comprehensive indexes. Compared with other factors, water has the biggest impact on the corrosion of slope protection net, followed by anion content. Total soluble salt has the moderate impact on the corrosion of slope protection net, and stray current has the moderate impact on the corrosion of mid-slope protection net. Comprehensive evaluation on the corrosive degree of soil samples indicates that the corrosion of upper slope is moderate, and the corrosion of mid-slope and lower slope is strong. Organic matter in soil is remarkably relevant to electric potential gradient. Available nitrogen, available potassium and available phosphorus are remarkably relevant to anions. The distribution of soil nutrient is indirectly relevant to slope type.

Excavating mountains is frequently ineluctable in constructing railways, roads, and water conservancy facilities. The railway construction in China calls for a large amount of mountain excavation due to the mountainous nature of the southwest region. It destroys original soil and vegetation, creating exposed rock slopes. The situation leads to landslide, water and soil loss, thus threatening the safety of railway transportation. Landslide is detrimental to road traffic, especially after the Wenchuan earthquake on May 12th, 2008. Landslide has become a widely distributed and serious seismic hazard^1^. When evaluating the key trunk roads of 4,243 km total length in Sichuan Province in 2008, we observed that 1,736 points of roadbed and slope retaining wall suffered serious seismic hazards, accounting for 39.76% of the total evaluated length. The direct economic loss caused by road damages was more than 58 billion yuan^2^,^3^. Global examples reveal that geological disasters after earthquake could last at least 10 years (Taiwan Jiji earthquake), and even up to 40–50 years (Japan Kanto earthquake)^4^,^5^. Gradient is the main factor influencing the disaster of earthquake^6^,^7^. Therefore, it is necessary to maintain road slopes and reinforce their stability. Plants play an irreplaceable role in slope protection and ecological landscape recovery^8^. Compared with common soil slopes, rock slopes, without accumulation of nutrient factors such as organic matter, nitrogen, phosphorus, and potassium, do not possess the soil environment necessary for the growth of vegetation. Due to factors of steep gradient and rain erosion, slope soil is easy to lose. The poor environment of the slope lacks the necessary conditions for plant growth and the soil on slope surface lacks the support stability^9^. Spraying base material on soil dressing to protect slopes is the slope ecological restoration is a technique commonly adopted in China. Artificial soil used for spraying is composed of broken rock, farmland soil, straw, compound fertilizer, water-retaining agent and binder (commonly used binders include Portland cement, organic glue, and asphalt emulsifier) based on the given proportion. The technical flow is: laying iron wire net on rocks first, then fixing iron wire net with rivet and anchor bolt, and spraying artificial soil containing seeds on slopes through special sprayers at last. No.14 rhombic metal net galvanized sufficiently is mostly employed, standard of meshes 5 cm × 5 cm and diameter 2 mm. The metal net can make the soil substrate form a lasting holistic plate on the surface of rock. The metal net will be corroded in soil for the soil itself is the electrolyte, and the corrosion degree depends on the feature of soil. Evaluation of soil corrosion factors is very important for assessment of metal mesh erosion caused by soil and eliminating hidden dangers of landslide.

Plant roots are believed to play an essential role in slope stabilization and erosion control^10^,^11^,^12^,^13^,^14^. To stabilize a slope against shallow landslides, vegetation can be used, as plant root systems fix soil against slippage^15^,^16^,^17^. Woody vegetation, particularly trees, can help prevent shallow landslides^18^. A kind of solid protection structure formed by vertical and lateral root system of plants plays the role of stake stiffening in soil. The development of root architectural pattern is genetically driven, and the soil environment plays a decisive role in these processes^19^. Corrosion to metal varies with different soil environment^20^. The corrosion extent of metals in soil can vary from quite fast dissolution to insignificant effects^21^. Artificial soil is significantly different from real “soil”. The formation of natural soil is the result of interaction between the external environment and various organisms for the tens of millions of years^22^,^23^,^24^. Whether a metal net combining rock slopes and artificial soil could function safely before woody vegetation forms a stable root and ecological system has a direct bearing on the development of natural economy and the improvement of life safety and ecological environment.

However, corrosion to metal leads to huge losses. Losses caused by metal corrosion accounted for 4% of the gross production value, according to the survey conducted by China on industries including the chemical machinery industry in the early 1980 s. Therefore, it is significant for economic construction to study corrosion mechanism and adopt protective measures. Soil is a complicated system composed of gas, liquid, solid, and microorganism. The metabolites of microorganisms can corrode materials, and stray current may also induce corrosion. Therefore, corrosion prevention to metal buried in soil is important. At present, research surrounding buried metal corrosion mainly focuses on (1) factors affecting corrosion to buried metal^25^; (2) metal-protection methods^26^,^27^; (3) methods of judging the metal corrosion degree^28^; and (4) corrosion of metal in different media^29^. However, all of the soils from the study are natural and have undergone sufficient soil-forming processes. However, there is no report on the corrosion in artificial soil of railways rock slopes.

Compared with other corrosive media, artificial soil is characterized by non-flowing, heterogeneity, seasonality, and regionalism. Metal corrosion in artificial soil is caused by electrochemical action between metal and the artificial soil. Apart from inborn factors, metal corrosion speed is determined by the surrounding environment. A variety of factors affect metal corrosion separately or in a combined way, such as moisture content, oxygen content, total soluble salt content, contents of anion and metal ion, pH value, soil microorganism^30^,^31^,^32^.

The question of how to make the artificial soil permanent in rock slopes has been a problem during the 30 years it has been in practice^33^. Shrubs or trees can not grow on some slopes after 10 years artificial nursing as a result of soil erosion. Soil on the surface of metal nets was washed away in some places. Some metal net fractured and lost all soil above and below it due to corrosion(Fig. 1). At present, the research of railway slope corrosion focuses on railway substation grounding grid corrosion, stray current corrosion generated by light rail, and corrosion of railway bridges^34^,^35^, tracks and other vehicle equipment^36^. There is currently no report about the corrosion of railway slope protection metal mesh. This paper researches the physical, chemical and electrochemical properties of the artificial soil on rock slope in the southwest China Sui-yu Railway, aiming to predict metal corrosion through evaluating the properties of the soil and provide theoretical and practical basis for soil ecosystem restoration and artificial slope artificial.

## Materials and Methods

### Study site characterization

The experimental site is located in the Sichuan hilly region near the Suining Railway Station (30°32′N, 105°32′E). Located in Centre of Sichuan Basin, the area is low mountains hilly, geological structure simple, fold flat. Water eroding, cutting and stacking forms eroded hilly landscape. The bedrock of whole territory is mainly limestone and its covering layer is mainly purple sand and mudstone. With poor integrity, the rock is a bulk and block structure. The study area belongs to the subtropical humid monsoon climate, with seasonal characteristics of early spring, hot summer, short autumn, and late winter. With rainfall abundant, light and heat resources rich, a period long frost-free (mean 285 days), climate mild, the annual average temperature 17.4 °C, the hottest month (August) average temperature 27.2 °C, extreme maximum temperature 39.3 °C. The coldest month is January (average temperature 6.5 °C), extreme minimum temperature −3.8 °C, and the annual average rainfall of 920 mm mainly concentrated in the July and August. The rain fall of spring, summer, autumn and winter vary greatly, And the proportion of rainfall each season throughout the year is accounted: 19–21%, 51–54%, 22–24% and 4–5%.

### Sampling design

The research site was a slope built in 2003 in Chongqing-Suining railway slope of about 45°, South-facing at the 1 kilometer range from Suining railway station in April 2012, the natural slope as control. Slope ecological restoration employed foreign dressing soil spurting technique eco-recovery. According to the railway side slope height, the slope could be classified as the upper slope, the middle slope, the lower slope (Fig. 2). Since the thickness of cut slope artificial soil was about 10 cm, in order to avoid soil metal mesh corrosion product pollution, we just took the soil 0–8 cm of the surface by stainless steel shovel. Each slope position set four replications, and each replication set 15–20 randomly sampling points. Each repeating was a mixture of 15–20 randomly determined by an S-shaped line sampling points. And its fresh weight was about 500 g. Brought samples by polyethylene zip lock bag back to the laboratory for processing. Dried the soil naturally, picked out gravel and plant & animal residues, crushed the soil by agate stick, and sifted except for coarse-grained by 20 mesh and 100 mesh nylon sieve.

### Analysis of soil samples

VICTOR4106 ground resistance tester produced in the Victory Instruments Company was used to determine of soil resistivity; Soil resistivity was measured in the field; Soil moisture was measured by drying method. DMP-2 portable digital mv/pH instrument with high input impedance was used to measure soil corrosion potential. Potential gradient and redox potential was measured by DMP-2 portable digital mv/pH, total soluble salts in soil by residue drying method, chloride ion content in soil by AgNO_3_ titration (Mohr method), Sulfate content of the soil by indirect EDTA titration, soil carbonate and bicarbonate by double indicator titration method, Soil organic matter by potassium dichromate oxidation heating method, soil alkali hydrolysable nitrogen by alkali solution diffusion method, total phosphorus in soil by H_2_SO_4_-HClO_4_ digestion Mo-Sb colorimetric method, the available phosphorus content in soil by the Olsen method (0.05 mol/L NaHCO_3_ solution as extraction agent), total potassium content in soil by sodium hydroxide melt - flame photometry method.

### Data processing

The experimental date systematized preliminarily. SPSS Statistics 20 was used to carry out average, standard deviation, one-way ANOVA (one-way ANOVA) and Person correlation analysis.

## Results and Discussion

Table 1 indicates electromechanical properties, anion and nutrient of soil at different slope levels. Corrosion potential, soil resistivity and east-west potential gradient of different slope levels is significant (P < 0.05). Oxidation-reduction potential of lower slope, mid-slope and natural slope is significant (P < 0.05). Potential gradient vertical with railway track, namely south-north potential gradient, shows as upper slope > lower slope > mid-slope. pH value of soil shows as lower slope > upper slope > mid-slope > natural slope. In terms of total soluble salt, natural slope is significantly higher than railway slope (P < 0.05). The total soluble salt contents of soil at the three levels of railway slope are all above 500 mg/kg, so the total soluble salt has the medium impact on metal corrosion. Organic matter content of soil is the highest at natural slope and the lowest at the lower slope (P < 0.05). Total nitrogen content is the highest at mid-slope and the lowest at upper slope; available nitrogen content is the highest at lower slope and mid-slope and the lowest at natural slope; total nitrogen content at upper slope and lower slope of railway is low, but available nitrogen content is high. This indicates that organic nitrogen mineralization speed at upper slope and lower slope is high. The available potassium content is the same with that of available phosphorous.

### Relationship of Slope Position and Soil resistivity

Soil resistivity, the index indicating conductivity, is the basic parameter for judging soil corrosiveness. Factors affecting soil resistivity include moisture content, total soluble salt content, pH value, soil texture, temperature, organic matter content, soil temperature, and tightness. Generally speaking, soil of low resistivity is of strong corrosiveness, vice versa. Judging soil corrosiveness by resistivity is the method commonly used by all countries. Table 1 shows each single index corrosiveness grade evaluation standard^37^,^38^.

In the light of the experiment results and standards of China (Table 1), if soil corrosiveness is solely evaluated through soil resistivity, soil at upper slope is of strong corrosiveness; soil at lower slope is of medium corrosiveness; and soil at mid-slope and natural slope is of weak corrosiveness.

**Figure 1:**
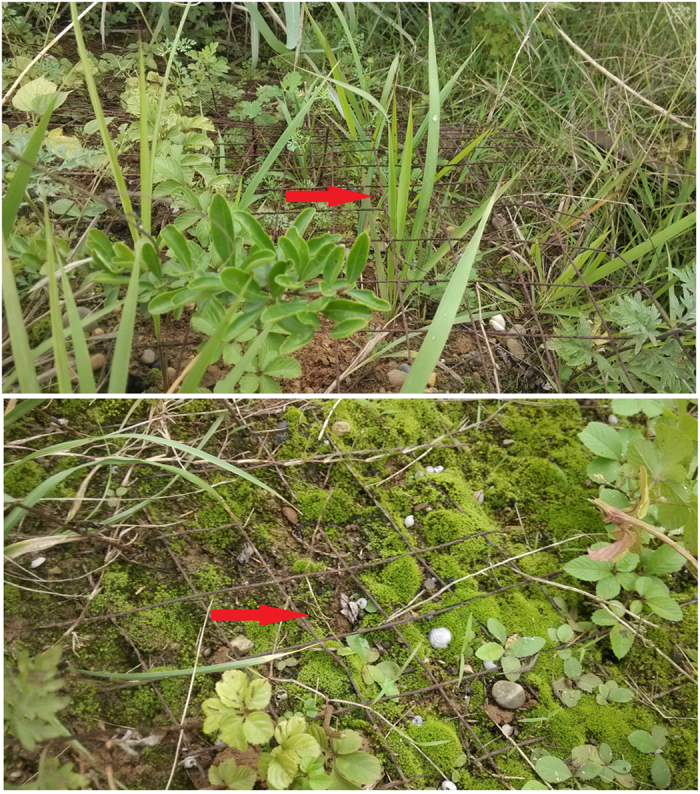
Bare metal mesh on the surface.

**Figure 2:**
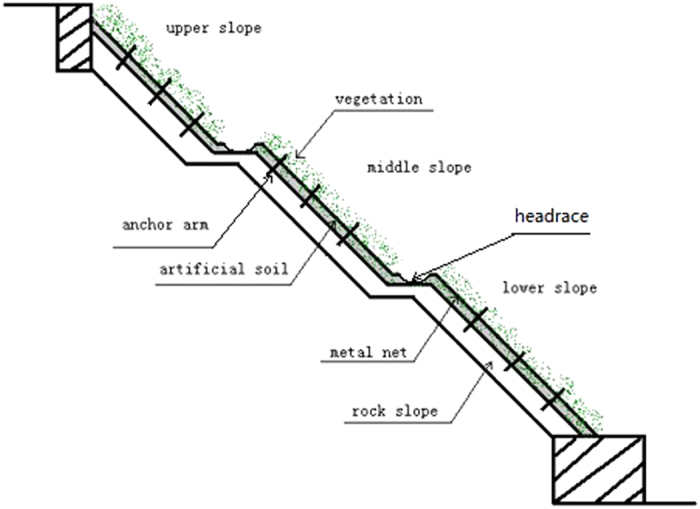
Foreign dressing soil spurting technique eco-recovery.

The fact that resistivity of soil at upper slope is remarkably lower than that at other parts of slope possibly attributes to the erosion of rainwater. Surface soil at upper slope flows to the middle slope with water, thus making metal slope protection net at upper slope close to surface soil. Some metal net is exposed and even suspended in the air (Fig. 1). Soil resistivity is measured at the site; the distance among piles is 3 m; and the depth of piles driven into soil is below 15 cm. Exposed metal net and peeled rust will have the given disturbance to measuring results. Therefore, it is unreliable to evaluate soil corrosiveness just by the index soil resistivity. In the comprehensive evaluation of corrosiveness, soil resistivity at upper slope will not be considered.

With high relative humidity, the year-round humid air in area of Sichuan leads to the fact that metal net exposed in air is more seriously corroded than that buried in soil^39^. Metal net exposed in air will suffer the decline in service life, thus making soil at upper slope unstable. Soil drain will make it difficult for plants, especially woody plants, to grow. Due to the deficiency of woody plants, it is hard to form root system at upper slope to solidify soil. Meanwhile, plant growth can also improve soil quality, and increase the content of humus in soil, which can preserve water and provide a favorable environment for the growth and reproduction of plants and animals, thus reducing the drain of soil. Therefore, it is advisable to sow more woody seeds at upper slope, add water-retaining agent continuously, and cover film for protection in the early period of construction so as to reduce rainwater’s erosion to soil at upper slope.

### Relationship of Slope Position and Electrochemical Properties

**Figure 3:**
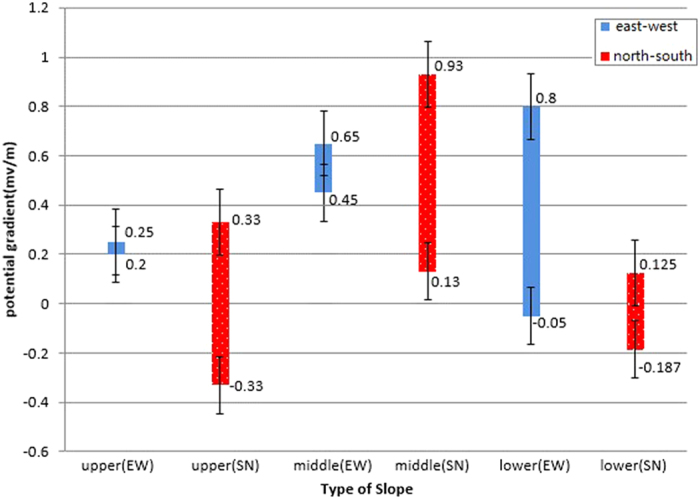
Floating range of potential gradient (EW) at mid-slope, upper slope and lower slope.

Corrosion potential is an important factor affecting the corrosion of slope protection nets at the three slope levels, and it has the biggest impact on upper slope (Table 2). In normal cases, corrosion potential does not change greatly in a given environment. A noticeable change is possibly caused by stray current. Stray current refers to the current that vehicles leak to roadbed and soil medium whilst using the public transportation system^40^,^41^,^42^. With the development of the transportation system, China has electrified the railway transportation system in a big way, and the corrosion to buried metal by direct current leaked from electrified railways cannot be ignored. Presently, soil potential gradient could be employed to judge whether soil contains stray current disturbance^43^. When surface soil potential gradient is below 0.5 mv/m, the degree of stray current is low; when potential gradient is ranging from 0.5 mv/m to 5.0 mv/m, the degree of stray current is moderate; when potential gradient is above 5.0 mv/m, the degree of stray current is high. Floating range of potential gradient (EW) at mid-slope, upper slope, and lower slope is shown as Fig. 3. In terms of the floating range, moderate stray current exists at the east-west and south-north directions of mid-slope, and the floating range of stray current is big at the south-north direction; Moderate stray current exists at the east-west direction of lower slope. Therefore, stray current is an important factor affecting the corrosion of metal nets at mid-slope and lower slope, especially mid-slope.

Generally the soil redox potential (Eh) above 400 mV means the capacity of oxidation, above 0–200 mv medium capacity of reduction, below 0 mV great capacity of reducing. The lower of soil redox potential is, the greater of soil microbial corrosion capacity on metal is^44^. According to the redox potential to predict the tendency of soil microbial corrosion is possible. The study found that soil oxidation -reduction potential of the three slope positions are more than 500 mv, and the corrosion rating is tiny. It illustrates the slope soil is in good ventilation conditions, not conducive to the anaerobic microbial corrosion in soil.

Previous research finds that the effect of soil pH on soil erosion is obvious. With pH fluctuating, the corrosion rate of metal materials is obvious influence. The pH value of soil is closely related to region and microorganisms in soil^45^,^46^,^47^. In general, the influence of soil pH on the corrosion of metal materials in slightly alkaline soil is unapparent. The soil in three kinds of railway slope is alkaline, so the influence of pH on metal net corrosion is weak.

As apparent from Table 3, the correlation analysis shows that there is a significant positive correlation in the redox potential and slope position (R^2^ = 0.858), a significant positive correlation in the corrosion potential and potential gradient (SN) (R^2^ = 0.755) and a significant negative correlation in the redox potential and pH (R^2^ = −0.724). Slope position has significantly positive correlation with the redox potential. It shows that the different slope positions have discrepant micro environment, soil microorganism and redox potential has a close connection^48^,^49^,^50^. Redox potential is significantly negatively related with pH^51^,^52^. The relationship shows that pH value and Eh value are not always changing synchronization relationship in soil redox process, but a negative linear correlation. Metal corrosion potential can represent relative ability of gain and loss electrons. While the corrosion potential and the potential gradient (SN) show a significant positive correlation, it may be due to the metal easily to lose electrons caused by the potential gradient.

### Relationship between Slope Position and Soil Anion

Soil total soluble salt content is closely related to soil corrosively. In general, the greater the amount of soil salinity, the smaller the resistivity of the soil, thus increasing the soil resistance. In soil electrolyte, not only the anion, and the range of variation, but the corrosion influenced mainly is carbonate, chloride and sulfate. In addition, the total soluble salt content in the soil through the influence of other factors indirectly affect corrosion, such as the effect of electrode potential in metal and soil oxygen solubility^53^.

The majority of soluble salt dissociation ions in soil are not directly involved in the electrochemical reaction, but through the soil resistivity to effect the metallic corrosion. The higher salt content of soil is, the stronger of soil conductivity, the stronger of soil erosion. Natural slope soil salinity content is significantly higher than the railway slope, probably due to the rich vegetation in natural slope conducive to soil, water and soil salinity conservation. The other reason may be the natural slope undergoing mature pedogenesis (soil parent material formed by the weathering of rocks), but the railway side slope soil consists of crushed rock fragments as matrix of “artificial soil” without soil adequate forming process, and the minerals are not released. In addition, deep soil salt ions of natural slope rise and gather in the surface soil by capillary action in the surface evaporation, leading to soil salt ions content in surface rising. The thickness of railway slope soil less than 20 cm results that surface soil cannot be replenished salt from deep soil.

Positive ions (such as K^+^, Na^+^, Ca^2+^, Mg^2+^, Al^3+^, and etc.) have no obvious impact on soil corrosiveness, but anions can significantly affect metal corrosion since they play a great role in the electrochemical process of corrosion. Cl^−^ can accelerate the corrosion to anode, so it is a kind of anion with the strongest corrosiveness; the higher Cl^−^ content is, the stronger soil corrosiveness is. SO_4_^2−^ can not only facilitate the corrosion to steel, but also cause the corrosion to some concrete materials^54^. 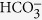 can also corrode iron. In a series of acid soil experiments, it is discovered that corrosion rate is in direction proportion to soil acidity^55^. Chloride ion and sulfate radical are the major ingredients of soluble salt, and can directly accelerate the cavitation of metal. Research reveals that corrosion weight loss of carbon steel in alkaline soil is almost in direct proportion to the addition of chloride ion and sulfate ion^56^,^57^. Researchers such as Lee found that SO_4_^2−^ could hinder the generation of corrosion, but boost the development of corrosion pits that have been formed^58^.

In the light of soil corrosiveness evaluation standards and also experiment results, chlorine ion contents in soil samples at each slope level are all above 100 mg/kg, so the soil is of strong corrosion. Contents of sulfate ion at upper slope and lower slope are above 200 mg/kg, below 500 mg/kg, so the soil is of moderate corrosion. Content of sulfate ion at mid-slope is below 200 mg/kg, so the soil is of weak corrosion. When soil medium contains 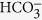 of relatively high concentration, 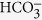 would participate in reaction and generate corrosion scale on the surface of metal electrode, thus slowing down corrosion reaction. With the increase of concentration, the scale may rupture suddenly, thus greatly accelerating the corrosion rate; with the continuous rise of 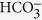 concentration, corrosion scale covers the surface of metal electrode, and corrosion rate again shows a tendency of deceleration^59^. It is found in the research that 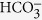 content is low in the soil, and thus has an insignificant impact on corrosion.

According to Table 4, the relevancy between slope level and anions in soil reveal that slope level is in significant positive correlation with chloride ion (R^2^ = 0.836), so is slope level with total soluble salt (R^2^ = 0.742).

This indicates that surface runoff and soil erosion are possibly the reasons for the change of total soluble salt in soil. The significant positive correlation between total soluble salt and chloride ion is possibly for the reason that total soluble salt is the bank of chloride ion, and the content of total soluble salt determines the content of chloride ion in soil solution. Hence, we can know that the difference of slope levels might lead to serious corrosion to parts of metal net.

### Relationship between Slope Position and Soil Nutrients

Organic matter, total nitrogen, available nitrogen, available phosphorus, and available potassium are the fundamental nutrients of soil, and affect soil quality and root system’s absorption of nutrients. Soil nutrients are the important factor that affects microorganisms in soil, so it is worthwhile for us to study whether there is correlation between soil nutrients and metal corrosion. Suiyu Railway was built in 2003, and this means that artificial soil has only undergone 9 years of organic matter accumulation. Due to the peculiarity of artificial soil, it is necessary to have a good understanding of nutrients in artificial soil.

The research reveals that soil at natural slope that has gone through the whole soil-forming process contains the largest quantity of organic matter. In the paper, organic matter content in soil at lower slope is the lowest. Due to the effects of weathering and surface runoff, soil nutrients would accumulate at mid-slope and lower slope, thus forming a thick humus layer. However, organic matter is likely to be decomposed by microorganisms because of small particles and poor stability of soil at lower slope. An investigation discovers that vegetation coverage and variety at mid-slope and lower slope are high, but homogeneity is low, which may lead to the uneven distribution of surface nutrients. The thick humus layer can preserve moisture, and soil organisms are active. All of these accelerate the decomposition of organic matters in soil.

Alkali-hydrolyzable nitrogen content of railway in upper slope, mid-slope and lower slope is higher than the natural slope, indicating that the organic nitrogen mineralization rate in railway slope is significantly higher than the natural slope. The smaller particle is, the more unstable soil structure is, the easier for microbial to decompose organic substances in aggregates, the bigger mineralized organic nitrogen pool is^60^,^61^. It is consistent with the results of the^62^, the small particles of soil aggregate content in railway slope is significantly higher than the natural slope. Therefore, we must take appropriate measures to improve fertilizer, organic matter and nitrogen content in the railway slope soil and enhance the sustainable utilization of soil. Available P and available K waste caused by surface runoff accounts for 77.27–99.79% of total loss in railway slope. Surface runoff may be a major driving force to available nutrient loss in the slope soil^63^,^64^,^65^.

As shown in Table 4, slope position and available phosphorus show significant positive correlation (R^2^ = 0.948), the same with correlation of slope position and available potassium (R^2^ = 0.898). It indicates that slope position affects the content of available phosphorus and available potassium in soil.

Gradient is an important factor affecting soil organic matter content and nitrogen enrichment^66^, the smaller of gradient, the greater of enrichment rate. For the effect of soil nutrient enrichment, nutrient loss is weakened, so the effect of slope position on soil organic matter content and total nitrogen enrichment is not obvious. The species and number of plant is divergent in different slope position, so is organic acids secreted by plant roots. Organic acid is conducive to the fixation of effective phosphorus and available potassium in soils. Therefore, slope position and available phosphorus, slope position and available potassium show a significant correlation.

To be clear about the relationship between soil nutrients and soil corrosiveness, it is necessary to analyze their relevancy. As Table 5 shows, oxidation reduction potential is in significant negative correlation with available nitrogen (R^2^ = −0.845), and in significant positive correlation with available phosphorus (R^2^ = 0.842) and available potassium (R^2^ = 0.980). Oxidation reduction potential reflects the oxidation-reduction quality, and is usually affected by some physical and chemical properties of soil, and in turn affects a series of properties of soil. Therefore, it is an important factor determining the conversion direction of soil nutrients^67^. Different oxidation-reduction qualities may lead to different status and effectiveness of nutrient factors. Hence, oxidation reduction potential is in significant correlation with available nitrogen, available phosphorus, and available potassium.

Apart from properties of metal, corrosion potential is also related to soil properties. Corrosion potential is in significant negative correlation with organic matters, which shows that organic matter could significantly affect corrosion potential. In addition, organic matters are also in significant negative correlation with potential gradient (SN) (R^2^ = −0.713) and sulfate ion (R^2^ = −0.671), which shows that organic matter content could also affect potential gradient (SN) and sulfate ion. Soil pH value is in significant negative correlation with available potassium (R^2^ = −0.728).

Available nitrogen is in significant negative correlation with total soluble salt and chloride ion while available phosphorus and available potassium is in significant positive correlation with total soluble salt and chloride ion. This shows that available nutrient content could significantly affect the quantity of total soluble salt and chloride ion in soil, and anions in soil are unfavorable for the accumulation and supply of available nutrients^68^. Total nitrogen is in significant negative correlation with sulfate ion, and in significant positive correlation with bicarbonate radical, which reveals that total nitrogen affects the contents of sulfate radical and bicarbonate radical. Plants demand little sulfate ion and bicarbonate ion, so most of them are free in soil, or absorbed by soil colloid. Bicarbonate ion is favorable for the accumulation of nitrogen in soil, and sulfate ion would reduce the effectiveness of nitrogen in soil. Therefore, appropriately increasing the content of available nitrogen and humus in soil is conducive to lowering soil corrosiveness.

### Comprehensive Evaluation of Corrosion

Soil is a kind of complicated system in terms of composition and property, and soil corrosiveness is the result of synergistic effect of various factors, so the comprehensive evaluation method is generally employed to evaluate the corrosiveness of soil. In the light of test methods of Code for Investigation of Geotechnical Engineering (GB50021–94) and Chinese soil corrosiveness test website, we can carry out comprehensive evaluation on soil corrosiveness grade according to the following standards: (1) evaluated to be weak corrosiveness if there is only weak corrosion and no moderate corrosion or strong corrosion; (2) evaluated to be moderate corrosiveness if there is no strong corrosion; (3) evaluated to be strong corrosiveness if there is one or two strong corrosions; and (4) evaluated to be serious corrosiveness if there are three strong corrosions and above.

According to soil resistivity, oxidation reduction potential, moisture content, salt content, pH value, and Cl^−^ and SO_4_^2−^ content, we conducted the comprehensive evaluation on the corrosion grade of soil samples from each slope level. The findings show that soil at each slope level is of strong corrosiveness.

## Concluding Remarks

Corrosion potential is an important factor affecting the corrosion to slope protection nets. Corrosion potentials at three slope levels are all below −200 mv, and have the strongest impact on the corrosion of metal net at upper slope. Potential gradient can be employed to judge the value of stray current in soil. Stray current is an important factor affecting corrosion of metal net at mid-slope and upper slope, especially mid-slope. Total soluble salt contents in soil at upper slope, mid-slope, and lower slope are all above 500 mg/kg, and have the moderate impact on the corrosion to slope protection net. Moisture content of soil is an important factor affecting the corrosion of metal net at mid-slope and lower slope, and has the strong impact on the corrosion to slope protection net. Soil at mid-slope contains the richest nutrients, and this indicates the frequent activities of microorganisms and the quick growth of plants there.

The research shows that corrosion potential, potential gradient, total soluble salt content, and moisture content are the major factors affecting soil corrosion at the three slope levels, and the corrosiveness of the soil is evaluated to be strong. Slope protection net suffers the severest corrosion at mid-slope, which offers the reference for the anti-corrosion design of railway slope protection net. Appropriately adding available nitrogen and organic fertilizer is conducive to reducing soil corrosiveness, helping plants grow, and finally stabilizing slopes.

## Additional Information

**How to cite this article**: Chen, J. *et al.* Impact of Soil Composition and Electrochemistry on Corrosion of Rock-cut Slope Nets along Railway Lines in China. *Sci. Rep.* **5**, 14939; doi: 10.1038/srep14939 (2015).

## Figures and Tables

**Table 1:** Soil electrochemical properties, soil anion and soil nutrient in different railway slope positions.

Soil sample	potential corrosion (mv)	Eh (mv)	potential gradient –EW (mv/m)	potential gradient –SN (mv/m)	Resistivity (Ω.m)	pH (25 °C)
Upper slope	−346c	530ab	0.23b	0b	18.93c	8.28a
Middle slope	−230b	510bc	0.55a	0.53a	51.28a	8.09b
Lower slope	−218a	540a	0.05c	0.03b	42.58b	8.29a
Nature slope	—	506c	—	—	50.25a	7.97c
Soil sample	Moisture (%)	total soluble salt (mg/kg)	Cl^−^ (mg/kg)	SO_4_^2−^ (mg/kg)	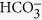 (mg/kg)	
Upper slope	10.48a	666.67b	272.60b	321.76a	80.83b	
Middle slope	14.73b	686.67b	275.66b	164.32b	87.25ab	
Lower slope	19.93c	683.33b	291.87b	294.88a	88.56ab	
Nature slope	17.41bc	1066.67a	368.89a	119.96b	89.06a	
Soil sample	OM (g/kg)	TN (g/kg)	AN (mg/kg)	AP (mg/kg)	AK (mg/kg)	
Upper slope	26.05a	0.46c	152.39b	12.98c	97.55c	
Middle slope	28.01a	1.08a	176.53a	15.65b	104.30c	
Lower slope	18.58b	0.87b	191.34a	21.34a	124.46b	
Nature slope	31.02a	0.83b	93.71c	25.99a	195.63a	

Notes: Column data marked with different superscripts mean significant difference (p < 0.05). Eh is the abbreviation for oxidation reduction potential. Potential gradient −EW is the abbreviation for potential gradient from east to west. Potential gradient −SN is the abbreviation for potential gradient from north and south. OM is the abbreviation for organic matter. TN is the abbreviation for total nitrogen. AN is the abbreviation for alkali-hydrolysable nitrogen. AP is the abbreviation for available phosphorus. AK is the abbreviation for available potassium.

**Table 2:** The correlation between railway slope position and electrochemical properties of soils.

	Corrosion potential	Oxidation reduction potential	Potential gradient (EW)	Potential gradient (SN)	pH
Slope position	−0.275	0.858**	0.218	−0.036	−0.589
Corrosion potential		−0.495	0.144	0.755*	0.405
Oxidation reduction potential			0.081	−0.314	−0.724*
Potential gradient (EW)				−0.020	−0.063
Potential gradient (SN)					0.385

Notes: Variables marked * are significant at the 0.05 level.

Variables marked * *are significant at the 0.01 level.

**Table 3:** The correlation between railway slope position and anion in soil.

	Total soluble salts	Cl^−^	SO_4_^2−^	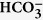
Slope position	0.742**	0.836**	−0.389	0.555
Total soluble salts		0.925**	−0.378	0.274
Cl^−^			−0.349	−0.186
SO_4_^2−^				−0.370

Notes: Variables marked * are significant at the 0.05 level.

Variables marked * *are significant at the 0.01 level.

**Table 4:** The correlation between railway slope position and soil nutrients.

	organic	Total N	alkali-hydrolyzable N	Effective P	Effective K
Slope position	0.005	0.434	−0.471	0.948**	0.898**
organic		0.130	−0.502	−0.043	−0.250
Total N			0.228	0.282	0.143
alkali-hydrolyzable N				−0.484	−0.778**
Effective P					0.867**

Notes: Variables marked * are significant at the 0.05 level.

Variables marked * *are significant at the 0.01 level.

**Table 5:** The correlation between soil nutrient and soil corrosivity.

	redox potential	corrosion potential	potential gradient (EW)	potential gradient (SN)	pH	total soluble salt	Cl^−^	SO_4_^2−^	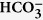
organic	0.345	−0.713*	−0.245	−0.671*	−0.496	0.308	0.145	−0.595*	0.222
Total nitrogen	0.142	−0.334	0.046	−0.527	−0.358	0.072	0.085	−0.717**	0.666*
available nitrogen	−0.845**	0.472	0.515	−0.107	0.596	−0.811**	−0.794*	0.262	0.063
available phosphorus	0.842**	−0.341	0.185	−0.044	−0.384	0.720**	0.858**	−0.335	0.421

Notes: Variables marked * are significant at the 0.05 level.

Variables marked * *are significant at the 0.01 level.
